# Anti-dipeptidyl-peptidase-like protein 6 encephalitis with pure cerebellar ataxia: a case report

**DOI:** 10.1186/s12883-022-02769-0

**Published:** 2022-07-01

**Authors:** Jing Lin, Min Zhu, Xiaocheng Mao, Zeqing Jin, Meihong Zhou, Daojun Hong

**Affiliations:** Department of Neurology, Dong’hu District, The First Affiliated Hospital of Nanchang University, No.17, Yongwaizheng Road, Nanchang, 330000 China

**Keywords:** Autoimmune encephalitis, Dipeptidyl-peptidase-like protein 6, Cerebellar ataxia, Antibody, Case report

## Abstract

**Background:**

Anti-dipeptidyl-peptidase-like protein 6 (DPPX) encephalitis is a rare autoimmune encephalitis. The clinical symptoms of anti-DPPX encephalitis are often severe, manifested as diarrhea/weight loss, central nervous system hyperexcitability and cognitive dysfunction.

**Case presentation:**

An 18-year-old boy was admitted for 1-week-long cerebellar symptoms including dizziness, unsteady gait and frequent vomiting. Magnetic resonance imaging (MRI) displayed no abnormal findings. However, autoimmune encephalitis panel revealed anti-DPPX antibody was positive in the serum. This patient completely recovered after immunoglobulin and corticoids therapy. In addition, repeat serum antibody test for DPPX was negative within one month.

**Conclusion:**

In addition to the classic triad, anti-DPPX encephalitis may manifest as mild and rare symptoms due to lower antibody titers. Fast identification of rare symptoms can help to quickly diagnosis and effective treatment.

## Background

Autoimmune encephalitis is a debilitating neurological disease mediated by antibodies to neuronal surface receptors or ion channels on neurological tissue [1]. In the past ten years, with the reports of various antibodies against the surface antigens of central nervous system neurons, autoimmune encephalitis has gradually been recognized by clinicians [2]. Diverse antibodies may lead to a variety of clinical manifestations including behavioral and psychiatric symptoms, autonomic disturbances, movement disorders, and seizures [2]. Anti-N-methyl-D-aspartate receptor (NMDAR) encephalitis, as the most frequently diagnosed encephalitis, often present with neuropsychiatric symptoms. However, morvan syndrome is the main feature of anti-contactin-associated protein 2 (CASPR2) encephalitis [2]. Anti-dipeptidyl-peptidase-like protein 6 (DPPX) encephalitis is a rare autoimmune encephalitis which was first described in 2013 [3]. DPPX is a regulatory subunit of Kv4.2 potassium channels and mainly expressed in the myenteric plexus, cerebellum, hippocampus and striatum [3]. Characteristically, most of these patients complain of diarrhea/weight loss, central nervous system hyperexcitability and cognitive dysfunction [3, 4]. Here, we describe a boy with only cerebellar symptoms and signs, a symptom rarely reported in anti-DPPX encephalitis. We think this case could enrich the symptom spectrum of this rare disease.

## Case presentation

An 18-year-old boy presented with 1-week-long dizziness, unsteady gait and frequent vomiting. He had unexplained low-grade fever a few days but no diarrhea before symptom onset.

The patient presented drunken gait and could not ambulate independently. A neurological examination showed horizontal nystagmus although extraocular movements appeared full in all planes. In addition, coordination movement tests demonstrated cerebellar ataxia, mainly on finger-nose testing, heel-knee-tibia test was uncoordinated, and the Romberg sign positive with eyes closed and opened. However, mental status, limb muscle strength and sensory system examination were normal in this patient.

**Fig. 1:**
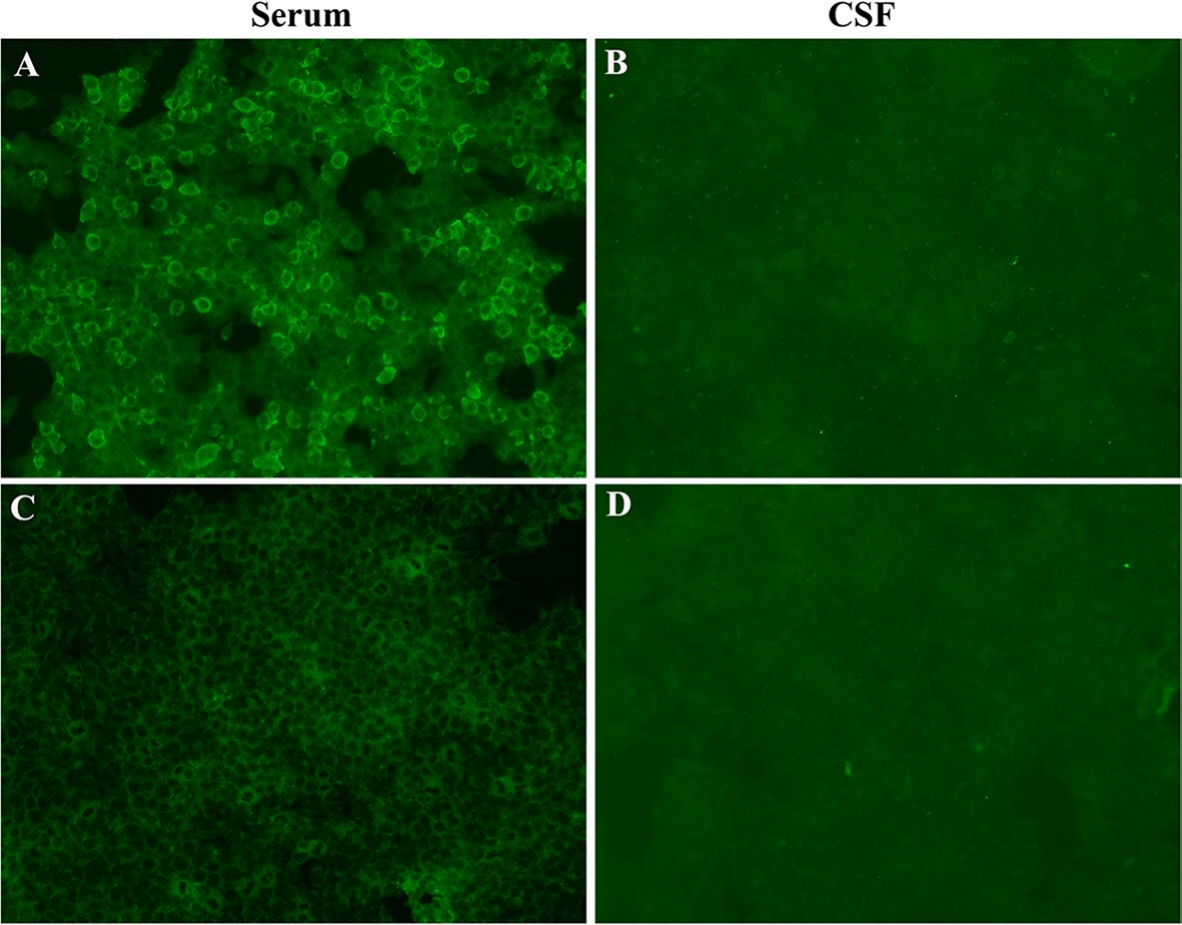
Positive reaction with transfected HEK293 cells expressing DPPX after incubation with the patient's serum (**A**) (titer 1:10) but negative with patient's CSF (**B**). The repeat (**C**) serum and (**D**) CSF antibody tests for DPPX were both negative after a 1-month follow-up visit

On hospitalization, there were no abnormal findings in laboratory analysis, brain magnetic resonance imaging (MRI), electroencephalogram and computed tomography imaging of the chest, abdomen. Cerebrospinal fluid (CSF) sample displayed normal white blood cells, protein and glucose levels. We sent an autoimmune encephalitis panel including serum and CSF samples, and anti-DPPX antibody was positive (1:10) in the serum through cell-based assay but CSF analysis revealed any abnormalities (Fig. 1 A-B). What’s more, other antibodies associated with tumors was negative.

The patient was treated with intravenous 500 mg methylprednisolone per day for 5 days, 240 mg per day for 5 days, 120 mg per day for 5 days, and followed by a slow tapering dose of prednisone over half year. In addition, he also received intravenous immunoglobulin (0.4 g/kg) for 5 days. When the patient was discharged from the hospital, the symptoms of dizziness and vomiting were significantly improved, and he was able to ambulate independently. Within one month, repeat serum and CSF antibody tests for DPPX were both negative (Fig. 1C-D). At follow-up 10 months after symptom onset, the patient remained clinically stable and had no cerebellar symptoms.

## Discussion and conclusions

Anti-DPPX encephalitis is a relatively rare autoimmune encephalitis. Up to now, no more than 50 cases have been reported [3–9]. The classic triad of clinical symptoms were diarrhea or weight loss, cognitive dysfunction and central nervous system hyperexcitability [4, 5], and 55% of patients had three classical symptoms in common (Table 1). Myoclonus, tremor, spasticity, rigidity, stiffness and hyperekplexia were the most common hyperexcitability in anti-DPPX encephalitis [4, 5]. Some rare symptoms have also been reported, including psychiatric symptoms (agitation, paranoia, hallucinations, anxiety, mutism, depression), brainstem disorders (eye movement disturbances, dysphagia, dysarthria, respiratory failure), dysautonomia (constipation, thermoregulation, urine retention, tachycardia), sleep disorder and seizures, occurring in 65%, 25%, 25%, 42.5%, and 12.5% of cases, respectively (Table 1). We presented a patient with pure cerebellar ataxia but no three classical symptoms. Although 57.5% of patients with anti-DPPX encephalitis displayed cerebellar ataxia, they all had other symptoms, and cerebellar ataxia was not the main clinical manifestation (Table 1). As far as we know, the case we presented was the first anti-DPPX encephalitis with cerebellar ataxia as the only symptom.

**Table 1:** The spectrum of symptoms in anti-DPPX encephalitis

**Case**	**Age** (years)	**Sex**	**Symptoms:** 1. Diarrhea or weight loss; 2. Hyperexcitability (myoclonus, tremor, spasticity, rigidity, stiffness, hyperekplexia; muscle cramps); 3. Cognitive dysfunction (memory loss, executive dysfunction); 4.Cerebellar ataxia (vertigo, ataxia, nystagmus, unsteady gait); 5. Psychiatric symptoms (agitation, paranoia, hallucinations, anxiety, mutism, depression); 6. Brainstem disorders (eye movement disturbances, dysphagia, dysarthria, respiratory failure); 7. Dysautonomia (constipation, thermoregulation, urine retention, tachycardia); 8. Sleep disorder; 9. Seizures;	**Reference**
1	61	Man	1,2,3,5	Boronat A et al. 2013 [3]
2	45	Female	1,2,3,5,8,9	Boronat A et al. 2013 [3]
3	58	Female	2,4,5	Boronat A et al. 2013 [3]
5	15	Man	2,3,4	Balint B et al. 2014 [6]
6	27	Man	1,2,4,7	Balint B et al. 2014 [6]
7	26	Man	1,2,3,4,7	Balint B et al. 2014 [6]
8	18	Man	1,2,3,4,6,9	Tobin WO et al [5]. 2014
9	57	Man	1,3,4,5,6,8	Tobin WO et al [5]. 2014
10	37	Female	1,5,7,8	Tobin WO et al [5]. 2014
11	36	Female	1,2,3,4,5,8	Tobin WO et al. 2014 [5]
12	51	Female	1,2,3,4,6,7	Tobin WO et al. 2014 [5]
13	75	Man	3,6,7	Tobin WO et al. 2014 [5]
14	61	Man	1,2,5,6	Tobin WO et al. 2014 [5]
15	70	Man	1,3,4,8	Tobin WO et al. 2014 [5]
16	63	Man	1,2,3,5,7,8	Tobin WO et al. 2014 [5]
17	39	Female	2,5	Tobin WO et al. 2014 [5]
18	52	Female	1,3	Tobin WO et al. 2014 [5]
19	66	Man	1,2,9	Tobin WO et al. 2014 [5]
20	24	Man	1,2,3,4,5,6,7,8	Tobin WO et al. 2014 [5]
21	13	Female	6,8	Tobin WO et al. 2014 [5]
22	49	Man	1,2,6,8	Tobin WO et al. 2014 [5]
23	53	Female	3,4,5	Tobin WO et al. 2014 [5]
24	55	Man	1,2,3,4,5,8	Tobin WO et al. 2014 [5]
25	46	Man	2,3,4,6	Tobin WO et al. 2014 [5]
24	40	Female	1,2,3,4	Stoeck K et al. 2015 [7]
26	52	Man	1,2,3,5	Stokin GB et al. 2015 [7]
27	36	Female	1,2,3,4,5,6	Hara M et al. 2017 [4]
28	52	Man	1,2,3,5,9	Hara M et al. 2017 [4]
29	68	Man	1,2,3,4,8	Hara M et al. 2017 [4]
30	67	Man	1,2,3,5,8,9	Hara M et al [4]. 2017
31	49	Man	1,2,3,5	Hara M et al [4]. 2017
32	57	Man	1,2,3,4,5	Hara M et al. 2017 [4]
33	45	Female	1,2,4,5,7	Hara M et al. 2017 [4]
34	57	Man	1,2,3,4,5,7	Hara M et al. 2017 [4]
35	69	Man	1,5	Hara M et al. 2017 [4]
36	72	Man	1,3,5,8	Zhou Q et al. 2020 [10]
37	77	Man	1,2,3,4,5,8	Deuel LM et al. 2020 [9]
38	53	Man	1,2,3,4,5,8	Ye L et al. 2020 [11]
39	44	Female	1,2,3,4,5,8	Mbonde AA et al. 2021 [8]
40	54	Man	1,2,3,4,5,7,8	Mbonde AA et al. 2021 [8]

In our patient, DPPX-IgG was detected positive in serum but not in CSF although we used the more sensitive cell-based assay rather than immunofluorescence. According to literature reports, in most patients with anti-DPPX encephalitis, both serum and CSF were positive for DPPX antibody [4, 5]. However, the serum DPPX antibody titers were significantly higher than the CSF DPPX antibody titer, which suggested that the positive rate of DPPX antibody in serum was higher than that in CSF [4, 5]. Combined with the clinical features, negative antibody profile of paraneoplastic syndromes, brain MRI, and cerebrospinal fluid characteristics, we excluded other diseases such as tumor, paraneoplastic syndrome and metabolic disease in this patient. Moreover, for acute or subacute cerebellar ataxia, we need to consider post-infectious cerebellar ataxia. Post-infectious cerebellar ataxia is an exclusive diagnosis and affects mainly younger children. Nussinovitch M et al. and Connolly AM et al. described in detail the clinical features of 39 and 73 children patients, respectively, and the mean age at presentation for these children was 4.8 ± 3.8 years and 7.4 ± 6.0 years [12, 13]. In addition, a prodromal varicella or mumps was noted in 31% or 20% children post-infectious cerebellar ataxia patients [12]. Of course, post-infectious cerebellar ataxia also occurs rarely in adults. Klockgether T et al. examined 11 adult patients (mean age, 40.7 ± 15.2 years) and 73% of the patients showed cerebellar oculomotor disturbances [14]. Our patient was 18 years old and had no prodromal varicella, mumps or cerebellar oculomotor disturbances so we didn’t consider this patient as post-infectious cerebellar ataxia. Furthermore, autoimmune encephalitis panel demonstrated positive DPPX antibody in serum, and the symptoms in this patient marked improved and DPPX antibody became negative after immunotherapy. Taken together, this patient should be considered as anti-DPPX antibody encephalitis. The antibody titer (1:10) in our patient was lower than other reported cases, which may explain the patient only has cerebellar symptoms.

The identified autoantibodies which present pure or primarily cerebellar ataxia can be divided into two categories: 1) specific autoantibodies, including anti-gliadin, TG2, and TG6 in gluten ataxia, and anti-Yo, Hu, CV2, Ri, Ma2 and Tr for paraneoplastic cerebellar degeneration. 2) nonspecific autoantibodies found in various neurological conditions, which assumed to have pathogenic roles in the cerebellar ataxia and include anti-VGCC, DPPX, LGI1, CASPR2, mGluR1, GAD65 and MAG [15, 16]. In order to reveal the cause of our patient, we sent autoimmune encephalitis antibody panel and paraneoplastic syndrome antibody panel which included the autoantibodies above. Finally, we found that only the DPPX antibody was positive, and the rest for pure or primarily cerebellar ataxia were negative.

In conclusion, it is important to recognize the uncommon symptoms of anti-DPPX encephalitis including cerebellar ataxia, psychiatric symptoms, dysautonomia, sleep disorder, and seizures in addition to the classic triad (diarrhea/weight loss, hyperexcitability and cognitive impairment). Fast identification of rare symptoms can lead to quickly diagnosis and effective treatment.

## Data Availability

Not applicable.

## Abbreviations

NMDAR: N-methyl-D-aspartate receptor
CASPR2: Contactin-associated protein 2
DPPX: Dipeptidyl-peptidase-like protein 6
MRI: Magnetic resonance imaging
CSF: Cerebrospinal fluid
TG: Transglutaminase
VGCC: Voltage gated calcium channel
LGI1: Leucine-rich glioma-inactivated 1
mGluR1: Metabotropic glutamate receptor
GAD65: Glutamic acid decarboxylase 65
MAG: Myelin-associated glycoprotein
